# Recent advances in the understanding and management of oropharyngeal cancer

**DOI:** 10.12688/f1000research.14416.1

**Published:** 2018-08-30

**Authors:** Ashley Hay, Iain J. Nixon

**Affiliations:** 1 Department of Head and Neck Surgery, Memorial Sloan Kettering Cancer Center, New York, USA; 2 Department of Otolaryngology Head and Neck Surgery, NHS Lothian, Edinburgh, UK

**Keywords:** Oropharynx, Squamous cell carcinoma, Robotics, Immunotherapy, IMRT

## Abstract

Oropharyngeal squamous cell carcinoma (OPSCC) is an increasing health problem in the developing and developed world. In recent years, there have been major changes in the treatment paradigms for OPSCC. This is because of a number of reasons: the understanding and discovery of a new viral etiology (the human papillomavirus [HPV]), changes in practice patterns owing to advances in radiotherapy, and then an organ preservation strategy with the increased use of chemotherapy. Next came the development of new surgical technologies and the emergence of a new treatment modality, immunotherapy. In this article, we discuss the evolution of OPSCC treatments, starting with the traditional tobacco era. Treatment paradigms then evolved during the organ preservation era, the HPV era, and the minimally invasive surgery era. We are currently in the immunotherapy era, with a number of new drugs becoming available both on trial and by approval for use in the clinical setting for head and neck cancer patients. We discuss a number of trials and the reasons behind attempts at both treatment escalation and treatment de-escalation.

## Introduction

### Anatomy and histology of the oropharynx

Oropharyngeal squamous cell carcinoma (OPSCC) refers to squamous cancers that develop in the posterior third of the tongue, tonsils, soft palate, and posterior pharyngeal wall ^1^. Within these subsites of the oropharynx, the tonsil and tongue base are the most common sites to be affected by carcinoma. These sites differ from the other subsites because they have a high density of lymphoid cells and show a strong association with human papillomavirus (HPV)-related squamous carcinoma. The strong predilection of HPV for the oropharynx is due to the microanatomy of the reticulated epithelium (epithelium with an immune system component) of the base of the tongue and tonsils ^2^. HPV infects the reticulated epithelium lining the deep tonsillar crypts. The deep crypts of tonsils are immune-privileged sites, which means they can tolerate the introduction of antigens without eliciting an inflammatory immune response. This results in inhibition of the effector function of the HPV-specific T cells and thereby facilitates immune evasion at the time of initial HPV infection ^3^.

In this article, we will discuss the evolution in understanding of OPSCC and describe how this has impacted on treatment options and research opportunities in this field.

## The evolution of oropharyngeal squamous cell carcinoma

### Tobacco era

Traditionally, oropharyngeal carcinoma was closely related to tobacco and alcohol exposure. The incidence of carcinoma in the oropharynx was similar to that in the other sites of the upper aero-digestive tract that all shared common exposure to the carcinogens in tobacco ^4^. There was also a high incidence of comorbidity in these patients from associated tobacco exposure conditions. Treatment was typically with surgery, as most tumors were locally advanced at presentation. Open approaches, such as mandibulotomy, were used to access the oropharynx. Long-term support of airway and feeding was often required in the form of a gastrostomy feeding tube and a tracheostomy. Radiotherapy was used post-operatively to optimize loco-regional control. With developments in the delivery of radiation and the recognition that primary non-surgical management was feasible, a move away from traditional open resection was seen. Two multi-center randomized controlled trials in 2004 also showed a survival advantage with the addition of post-operative chemoradiotherapy in high-risk patients ^5, 6^. Radiation-based approaches were adopted more commonly with surgery reserved for salvage. A report of the outcomes and complications comparing surgical and radiotherapy-based approaches in 2002 showed that while both approaches had similar survival and local control rates, surgery was associated with an increased rate of complications ^7^.

### Organ preservation era

Landmark trials fueled the enthusiasm for organ preservation approaches in head and neck cancers and built upon increasing radiation treatment experience and expertise. In 1994, the Veterans Affair study reported outcomes for locally advanced laryngeal carcinoma treated with induction chemotherapy followed by radiotherapy (in responders) compared with total laryngectomy. The results of this study confirmed that a non-surgical organ preservation approach could be adopted, which achieved similar oncological outcomes but avoided laryngectomy in over 60% of cases. A further study in locally advanced laryngeal carcinoma by Forastiere *et al*. showed a survival benefit in the use of concomitant chemoradiotherapy (chemotherapy administered during radiation) over induction chemotherapy or radiation alone ^8^. These results were extrapolated to other head and neck sites and provided the initial support for organ preservation strategies. The first major OPSCC-specific trial was authored by the GORTEC group. They randomized 266 patients with OPSCC to concomitant chemoradiotherapy or radiotherapy alone ^9^. An overall survival at 3 years was reported as 51% in the chemoradiotherapy group versus 31% for the radiotherapy-alone group ^9^. At 5 years, a 6.6% overall survival benefit with the addition of chemotherapy was reported ^10^. This has subsequently become a standard therapy for loco-regional advanced OPSCC and was supported by a robust meta-analysis ^11^.

### Improvements in radiation

Further interest in radiation treatments for OPSCC was stimulated by advancing technologies in the delivery of radiotherapy. Intensity-modulated radiotherapy treatment (IMRT) was introduced in the early 2000s. This was a new radiotherapy technique aimed at reducing the comorbidity of treatment by allowing the oncologist to manipulate radiation dose in a way that would increase accuracy and reduce the dose to bystander structures. Specific potential advantages of this targeted method of delivering radiation include avoidance of high-dose exposure to the parotid glands to minimize xerostomia ^12^ and to the pharyngeal constrictors in an attempt to minimize swallowing dysfunction ^13^.

### Human papillomavirus era

The next major development was the discovery of a viral etiological agent in the carcinogenesis of OPSCC. An association of HPV with carcinomas of the tonsil and base of the tongue emerged in the early 1990s, with the identification of HPV DNA in tumor cell nuclei and viral oncogene transcription in tonsillar carcinomas ^14^. This coincided with changes in the incidence and demographics of OPSCC ^15^. The incidence of tonsillar carcinomas in the United States increased by 1.9% and 2.7% per year from 1973 to 1995 in Caucasian and African-American men, respectively, while all other oral sites remained constant. The impact and relevance of these discoveries were crystallized by Kian Ang *et al*. in 2010 ^16^. They reported a retrospective analysis of the association between tumor HPV status and survival among patients with stage III or IV OPSCC. In 323 patients, HPV was detected in 206 tumors (63.8%). The HPV-positive tumors had a better 3-year overall survival (82.4% versus 57.1%, *p*<0.001) and, after adjustment for age, race, tumor and nodal stage, tobacco exposure, and treatment assignment, had a 58% reduction in the risk of death (hazard ratio 0.42; 95% confidence interval [CI] 0.27–0.66). The effect of HPV status on outcome has now been shown in many series throughout the world. A meta-analysis of studies worldwide reporting on 5,681 patients showed the prevalence of HPV tumors was 22% and this was associated with an improved survival, with HPV-positive tumors having a 68% reduced risk of death compared to HPV-negative tumors (hazard ratio 0.42, 95% CI 0.27–0.57) ^17^.

The proportion of OPSCC with detectable HPV DNA on testing approached 50% in a number of series ^14^, but the rate does vary by country. A UK series has shown up to 70% of oropharyngeal tumors are HPV related ^18, 19^. The HPV16 subtype is the most common and is found in 90% of tumors. The other high-risk subtypes 31, 33, and 18 have also been identified ^14^. In an East-Denmark study between 2000 and 2010, Garnæs *et al*. found 58% of tonsillar OPSCC were HPV-related tumors and 51% in the base of the tongue were also HPV related ^20, 21^. However, high-risk HPV DNA showing evidence of transcription is infrequently seen at other sites in the head and neck and at the subsites of the soft palate and posterior pharynx ^18^. A multicenter cross-sectional retrospective study in the UK, however, has confirmed an increase in OPSCC cases but also showed that the percentage of tumors that were HPV related remained static ^22^.

This difference in anatomical location has allowed for the investigation of population-level data ^23^. An analysis of Surveillance, Epidemiology, and End Results (SEER) data from 1973 to 2004 with patients deemed likely to be HPV positive based on if the tumor’s location was in the tonsil or the base of the tongue showed patients were younger (61.0 versus 63.8 years, *p<*0.001) ^23^. A series of 193 patients, using DNA PCR to test for HPV, showed that HPV-positive tumors were more likely to affect patients younger than 55 years old ^24^. Age was recognized as an important distinction between HPV-positive and -negative tumors in a study from Sweden ^25^. Patients with HPV-related cancers were younger (mean age of 59 years [range 42–78] versus 66 years [range 45–89]).

Additionally, USA SEER data indicated that men have a higher incidence of OPSCC than do women and that blacks are more commonly affected ^23^. However, when likely HPV-positive tumors are examined, they were seen to have a higher incidence in white men and an increasing incidence in men in all other races ^23^. There was also a correlation between the educated middle class and HPV-positive cancers ^24^.

Other differences between HPV-positive and -negative tumors have also been seen. Tobacco has been identified as a head and neck cancer risk factor; it also acts synergistically with alcohol ^26^. A 25,500-patient multicenter study found that tobacco is a major risk factor for head and neck and oropharynx carcinoma ^27^. Patients with HPV-positive tumors have been shown to be non-smokers more often ^28^, and the overall tobacco use is lower compared to that in HPV-negative patients. However, smoking is still a risk factor but has less of an impact in HPV-related cancers. Patients with HPV-positive tumors have about 30% non-smokers in their group compared with less than 5% in the HPV-negative groups ^29^. This relationship has been seen in a number of studies ^30^.

The majority of studies that supported the use of chemoradiotherapy in patients with OPSCC were performed in the 1990s and early 2000s. At this time, the link between HPV and OPSCC was being uncovered. Unfortunately, it has not been possible to retrospectively analyze the patients in these studies to understand what proportion of these tumors were HPV related.

Other differences are also seen in the clinical presentation of HPV-related OPSCC. They tend to have smaller primary T stage ^31^ tumors with larger cervical nodal disease ^32^. The nodal disease is often also cystic. However, despite presenting with advanced nodal disease, which is traditionally thought to be associated with poor outcome, they are associated with an improved prognosis. This means HPV status could have acted as a confounder in studies that have not stratified for HPV status. The recurrence and failure rates are lower in the doubly positive HPV/p16 tumors compared to the HPV/p16-negative tumors ^33^. Other important differences in risk factors for HPV-related carcinoma compared to non-HPV-related OPSCC are the male preponderance and sexual exposure ^34^. Recognition of the major differences in risk factors, demographics, clinical behavior, and response to treatment of HPV-positive compared to HPV-negative tumors ^16, 35^ may allow more tailored treatment.

### Treatment trends and outcomes

In a study using the National Cancer Database (NCDB), a North American governmental database of cancers and treatments, the trends in the treatment modalities used in OPSCC were examined ^36^. In an analysis of 43,983 patients between 1998 and 2009, the number of patients receiving chemoradiation increased from 22% of all patients in 1998 to 61% in 2009. This has been accompanied by a concurrent decline in the percentage of patients receiving surgery, from 41% in 1998 to 31% in 2009. This coincides with the increasing incidence of OPSCC and increased HPV-related disease.

In recent studies using modern IMRT techniques, outcomes of chemoradiation for OPSCC have been excellent. In a European study ^37^ analyzing the survival and toxicity outcomes with primary IMRT, 186 patients received IMRT with 90% of loco-regionally advanced disease receiving concurrent chemotherapy or cetuximab. The estimated 3-year overall survival (OS), disease-free survival (DFS), and disease-specific survival (DSS) rates were 77.2% (70.5–83.9), 72.3% (65.4–79.2), and 80.2% (74.1–86.3), respectively. Estimated 3-year OS, DFS, and DSS rates for HPV-positive patients were 90.9% (85.2–96.6), 87.9% (81.4–94.4), and 91.8% (86.3–97.3), respectively.

Despite the excellent oncological results achieved with this approach, the changing demographic of patients with OPSCC and the improvement in survival means new challenges are being seen. The complications and sequelae of treatment are now more pertinent because patients are younger, more likely to be cured, and more likely to live longer.

Bird *et al*. ^37^ reported that three (1.6%) patients died during or within 30 days of radiation completion, 74 (40%) were admitted at least once during their radiotherapy, and 76% needed a feeding tube either as a supplement to their oral intake or to meet their complete nutritional requirements. However, long-term toxicities are likely to be more important in understanding which treatment is best for patients, as all treatment approaches are associated with acute toxicity.

### Minimally invasive surgery era

Our understanding of OPSCC etiology and its relationship with HPV infection has evolved at a time when non-surgical organ preservation approaches in head and neck oncology have replaced more traditional open surgeries with post-operative radiotherapy. However, over a similar time frame, significant progress has been made in surgical technology. Advances in optics and instrumentation have made a number of options available for the removal of head and neck cancers via a trans-oral endoscopic route. Trans-oral laser techniques have long been used in the larynx, and similar approaches have been adapted to use in the oropharynx ^38^. However, access can be challenging, particularly to the posterior tongue base. Since the US Food and Drug Administration’s (FDA) approval of trans-oral robotic surgery (TORS), robotic surgery has offered a minimally invasive approach for head and neck tumors. The da Vinci robot (Intuitive Surgical Inc, Sunnyvale, CA, USA) began its development in urology and cardiac surgery. In 2005, Melder and McLeod reported its first use in head and neck surgery when they performed a resection of a vallecular cyst ^39^. In 2006, Weinstein *et al*. at the University of Pennsylvania reported the robot’s first application to head and neck malignancy ^40^; their group conducted most of its early research and coined the term TORS. Subsequently, in 2009, the FDA approved its use in the head and neck, and TORS is now utilized worldwide.

Trans-oral surgery has emerged as an approach that offers an alternative to open surgery and primary non-surgical treatments and is being used in the staging/diagnosis of unknown primary patients ^41^. The advantages of TORS are the ability to operate without line-of-sight restrictions that limit other trans-oral endoscopic or microscopic approaches ^42^. The approach offers a consistent approach in which the pharyngeal muscle constrictors are removed to provide a deep margin. It allows tumors that would normally demand a pharyngotomy or mandibulotomy to be resected. Additionally, it involves instruments with six degrees of freedom, motion scaling, instrument stabilization, and tremor reduction ^43^. Precise three-dimensional visualization is aided by binocular and magnified endoscopic vision. Trans-oral microsurgery offers a different philosophy which was initially popularized by Steiner ^44^, with some centers reproducing excellent results with the technique ^38, 45^. A bespoke tumor resection is performed in which the deep margin is assessed using the microscope and tissue characteristics during resection. The tumor may be carefully divided to allow removal and resection in different sections. Careful histopathological analysis is required to understand where the true margin of the tumor is.

Trans-oral laser microsurgery (TLM) and TORS have demonstrated excellent oncological results for many indications and subsites, mostly in single institutional studies and oropharyngeal disease ^46, 47^. TORS has also shown promising functional outcomes with appropriate adjuvant therapy ^48^. A multi-institutional study of TORS has recently reported a 3-year survival rate of 92.5% and a 3-year recurrence rate of 88.8% ^49^.

### Immunotherapy era

The most recent development in the treatment of head and neck cancer is immunotherapy. Significant progress has been made in the application of immune checkpoint inhibitors such as nivolumab, pembrolizumab, durvalumab, atezolizumab, and avelumab. The checkpoint inhibitors pembrolizumab and nivolumab are FDA approved in the recurrent and metastatic setting and have an established paradigm for use. The majority of current studies are assessing drugs in the end-stage setting and also using a combination of treatments ^50^. However, there are currently 16 trials exploring the use of these drugs in the primary setting with curative intent.

A majority of these drugs act on the PD1 (programmed cell death protein)/PDL1 axis. This is applicable to head and neck and OPSCC because the oropharynx is known to be an immune-privileged site. The reticulated epithelium is known to express the immune checkpoint ligand PDL1, and the resulting reduction in cytotoxic T cell response has been linked to persistent HPV infection at these sites ^51^.

## Current treatments and trials

### Current treatment options

Despite major differences in the risk factors, demographics, clinical behavior, response to treatment, and molecular patterns of HPV-positive compared to HPV-negative tumors ^16, 35^, the recommended treatment options are still the same, unless the patient is on a trial. Treatment is decided using the Tumor, Node, Metastasis (TNM) stage, the patient’s preferences, the patient’s co-morbidities, and the physician’s experience ^26^. For loco-regionally advanced oropharyngeal cancer, dual modality treatment with either trans-oral surgery and post-operative radiotherapy with or without post-operative chemotherapy or concurrent chemoradiotherapy is usually offered. In cancers of the tonsil and base of the tongue, chemoradiotherapy is used more frequently ^52, 53^. Early stage disease can be treated with single modality treatment, such as surgery or radiotherapy alone.

The long-term toxicities following chemoradiotherapy have been questioned ^54^ and have provided impetus and enthusiasm for trans-oral resection as a primary treatment for these tumors ^55^. There are no randomized trials comparing trans-oral approaches versus IMRT, and the majority of the literature is limited to uncontrolled reports ^56^. Comparable oncologic outcomes with TORS compared to IMRT have been reported in these studies and functional outcomes *may* be superior. However, current follow-up is relatively short, and the TORS studies include patients with earlier-stage OPSCC on average compared to comparable IMRT studies.

### Current trials in OPSCC

The use of surgery in HPV-positive oropharyngeal cancer and the application of minimally invasive techniques to avoid or reduce required doses of adjuvant treatment have become important areas of study ^57^. Trans-oral laser surgery (TLS) was first popularized by Steiner in Germany ^44^. There has been increasing experience with TLS, but its use for oropharyngeal tumors has been limited to a few high-volume centers in the USA ^58^ and European units in the UK, France, and Germany ^57^.

De-escalation based on surgical resection and the neck stage is being assessed in HPV-positive tumors In the Sinai Robotic Surgery Trial (NCT02072148). Another study, conducted at the University of Pennsylvania, is employing robotic surgery to de-escalate adjuvant treatment. Reduced treatment to the primary tumor bed in fully resected tumors (NCT02225496) is being investigated. The Eastern Cooperative Oncology Group (ECOG) 3311 study is a large, randomized, multicenter study comparing normal-dose post-operative radiation with low-dose treatment (NCT01898494) following minimally invasive surgery; patients are stratified post-operatively, with low-risk patients being observed, high-risk patients receiving chemoradiotherapy, and intermediate-risk patients being randomized to normal or low standard-dose radiotherapy.

The PATHOS trial (Post-operative Adjuvant Treatment for HPV-positive Tumors) is a UK-based multicenter randomized trial. It is comparing post-operative treatment for HPV-positive disease. With pathological information from the surgical resection, patients will be classified as low, intermediate, or high risk. Patients who are low risk will be observed without adjuvant treatment. Patients who are intermediate and high risk will be randomized to an adjuvant treatment. The intermediate-risk group will be randomized to either high-dose or low-dose radiation and the high-risk group to standard radiotherapy either with or without chemotherapy (NCT02215265).

The ADEPT trial (Post-Operative Adjuvant Therapy De-intensification Trial for HPV-related, p16+ Oropharynx Cancer) examined patients with fully excised HPV-positive tumors and randomized patients to either radiotherapy or radiotherapy plus cisplatin (NCT01687413). It is now closed to accrual. The use of post-operative docetaxel with hyper-fractioned IMRT is being investigated in another trial following minimally invasive surgery (NCT01932697). In the Canadian ORATOR trial, a phase II randomized trial, patients with early stage OPSCC were randomized to receive radiotherapy or trans-oral robotic surgery with neck dissection (NCT01590355). In this “best of” randomized study, patient-reported swallowing function over the first year will be investigated following allocation to either IMRT or TORS in patients with early stage OPSCC (NCT02984410).

There are a number of other trials that are using modification of the standard radiotherapy or chemotherapy techniques. In HPV-positive oropharyngeal tumors, cisplatin alternatives are being trialed, as they are thought to have less toxicity. With concurrent radiation, epidermal growth factor receptor (EGFR) therapies are being investigated. Cetuximab is the most common agent. It is a monoclonal antibody that targets the EGFR extracellular ligand-binding domain on the cell surface.

The Radiation Therapy Oncology Group (RTOG) are conducting a randomized trial of cisplatin versus cetuximab with radiation (NCT01302834). De-ESCALaTE (Determination of Cetuximab Versus Cisplatin Early and Late Toxicity Events) is a UK trial comparing either cisplatin or cetuximab with radiation and using toxicity as a primary outcome (NCT01874171). Also, the Australian Trans-Tasman Radiation Oncology Group (TROG) have a trial comparing cetuximab to cisplatin with radiotherapy (NCT01855451). An additional study is investigating cetuximab with pre- and post-treatment biopsies, which will then be compared to a historical series of cisplatin-treated patients (NCT01663259).

Reducing or modulating the radiation dose following induction chemotherapy is another strategy. In a RTOG trial, depending on how patients respond to induction chemotherapy with paclitaxel, cisplatin, and cetuximab, cetuximab combined with either low- or standard-dose IMRT will be administered (NCT01084083). The Quarterback Trial (NCT01706939) compares a decreased radiation dose with weekly carboplatin to a standard radiation regimen with weekly carboplatin following induction chemotherapy in patients with a good response. Also, the OPTIMA trial is investigating de-intensifying treatment based on response to induction chemotherapy. Using nab-paclitaxel and carboplatin as an induction regimen, the investigators will administer a response-based therapy. Chemotherapy with high- or low-dose radiation or just radiation alone is being investigated in stage III or IV HPV-related OPSCC (NCT02258659). Paclitaxel and carboplatin used as an induction therapy with concomitant paclitaxel in tumors of HPV-positive patients is another regimen under investigation (NCT02048020).

In HPV-positive patients, reducing the radiation dose is another approach. Reduction of the dose of IMRT to 54–60 Gy while simultaneously administering weekly intravenous cisplatin will precede surgical resection of any clinically apparent residual tumor or neck disease (NCT01530997).

A randomized trial of HPV-positive oropharynx tumors using a reduced dose of IMRT treatment with randomization to radiotherapy alone or concomitant cisplatin presents an additional approach (NCT02254278). Treatment de-intensification is being investigated in another study alongside cisplatin chemotherapy with a reduced radiation dose, with the experimental arm’s dose decreasing from 70 Gy to 63 Gy (NCT01088802).

There are other new agents and immune therapies currently being trialed. Ribavirin is being evaluated as part of a phase I trial. It is a drug that is used in the treatment of hepatitis C, and it is being used with afatinib (a tyrosine kinase inhibitor) as an induction chemotherapy agent with weekly carboplatin/paclitaxel dose (NCT01721525).

Immune therapy and vaccines are thought to offer an additional modality of treatment because foreign viral antigens could be amenable to targeted therapy. The local presence of HPV16-specific T cell immunity found in HPV16-induced SCC suggests a basis for this ^59^.

The use of T cells as an autologous transfusion is a process termed adoptive immunotherapy. Other highly immunogenic tumors such as melanoma have responded to this type of therapy ^60^, and it is now being investigated in the head and neck.

Antitumor vaccines are a different immune-modulating therapy aiming to stimulate a host’s immune system in the treatment of cancer. In a phase I trial, recombinant *Listeria monocytogenes* is the base for a vaccine that has been modified to express HPV16 targets (REALISTIC trial). This trial was closed early.

In contrast to efforts to de-escalate treatment of HPV-positive low-risk tumors, HPV-negative tumors and some HPV-positive tumors in patients with a smoking and alcohol history are deemed high risk. In these patients, there may be benefit in escalating treatment to improve outcomes. In the COMPARE trial (UKCRN Study ID: 18621), additional treatments in conjunction with standard chemoradiotherapy are being investigated compared to standard chemoradiotherapy. Patients are randomized to either the standard or receive surgery followed by chemoradiotherapy, more chemotherapy as induction (docetaxel, cisplatin, and 5-fluorouracil), a higher dose of radiotherapy as part of their chemoradiotherapy, or durvalumab with chemoradiotherapy.

## Changes in testing and staging

### Testing for human papillomavirus

There has been controversy regarding the different techniques and biomarkers used to determine whether a tumor is related to HPV infection. Sustained and persistent high-risk HPV E6/E7 viral oncogene expression is essential for a HPV-driven malignant tumor ^53^. The detection of HPV E6/E7 mRNA transcripts correlates with cellular genotoxic damage and gene expression changes that are the hallmarks of cancer. However, the detection of mRNA in the clinical setting is difficult and expensive ^61^. Another approach is using p16 as a surrogate for HPV infection and could utilize the cheaper and more available immunohistochemistry stains. However, when a number of these different assays were tested, only the RT-PCR assay for HPV16 E6*I mRNA developed specifically for formalin-fixed paraffin-embedded (FFPE) material was able to accurately classify samples ^62^. Using the different biomarkers for prediction of outcome, the doubly positive p16/HPV DNA test had the best predictive ability, with p16-positive/HPV-negative tumors having a prognosis closer to more typical non-HPV-related tumors ^33^. Therefore, it has been proposed that the combination of p16 immunohistochemistry and the detection of HPV DNA by PCR is required ^63^. Other testing methods for HPV infection involve the use of saliva samples from swish and spit specimens ^64^. This may provide a potential screening test for OPSCC or may replace the need for formal biopsy ^65^.

### Union for International Cancer Control/American Joint Committee on Cancer Tumor, Node, Metastasis staging system eighth edition

Another recent change has been the introduction of an updated staging system for OPSCC. The eighth edition of the Union for International Cancer Control/American Joint Committee on Cancer (UICC/AJCC) TNM system was introduced into clinical practice in January 2018. However, the current UK policy is to not use the TNM eighth edition for treatment allocation. The data used to revise the system are largely retrospective and therefore treatment de-intensification should currently only occur in the context of an appropriate clinical trial.

OPSCC has been divided into p16-positive, high-risk HPV-associated tumors and p16-negative tumors ^66^. In the T staging category, the Tis and T4b stages have been removed from HPV-related tumors. There has been the introduction of separate clinical and pathological N staging systems. The clinical N staging has been simplified, with the N1 stage now including all patients with ipsilateral nodes less than 6 cm. This will downstage patients who previously had T2b neck disease in the seventh edition. N2 disease includes patients with contralateral or bilateral disease, and N3 is for patients with a neck mass of more than 6 cm. In the pathological staging system, N1 disease is fewer than four positive nodes, N2 is more than four positive nodes, and N3 has been removed.

## Conclusion

OPSCC is an evolving field. For many years, surgery and radiation therapy were the only options for treatment in the majority of cases. While this is still true, a number of new and exciting options are becoming available. Advances in radiotherapy techniques and the introduction of IMRT allow more accurate dose delivery to the tumor while sparing surrounding structures to minimize side effects. Improvements in our understanding of the biology of HPV-related disease have led to attempts to de-intensify treatment regimens to further reduce the morbidity of therapy in low-risk patients. Surgical techniques have incorporated state-of-the-art technology, such as new robotic and laser systems. These advances offer the potential to reduce the long-term morbidity associated with chemoradiation and may also play a role in the intensification of treatment for the highest-risk HPV-negative patients. There is also a whole new modality, immune therapy, with new drugs receiving FDA approval in the setting of recurrent/metastatic head and neck cancers. It seems likely that, over the next decade, we will see a move away from standard chemoradiotherapy techniques to a more individualized approach based on the patient and tumor factors in an attempt to optimize oncological outcome while minimizing morbidity for a growing number of patients with OPSCC. Other changes have emerged as our understanding of HPV-related tumors has improved. This is particularly shown with the introduction of the new TNM eighth edition staging system.
